# Inter-nesting, migration, and foraging behaviors of green turtles (*Chelonia mydas*) in the central-southern Red Sea

**DOI:** 10.1038/s41598-023-37942-z

**Published:** 2023-07-11

**Authors:** Lyndsey K. Tanabe, Jesse E. M. Cochran, Michael L. Berumen

**Affiliations:** Division of Biological and Environmental Science and Engineering, Red Sea Research Center, King Abdullah University of Science and Technology, 23955‑6900 Thuwal, Kingdom of Saudi Arabia

**Keywords:** Animal migration, Behavioural ecology

## Abstract

Sea turtles are migratory with nesting and foraging areas in distinct and often widely separated habitats. Telemetry has been a vital tool for tracking sea turtle migrations between these areas, but tagging efforts are often focused on only a few large rookeries in a given region. For instance, turtle tagging in the Red Sea has been focused in the north of the basin. We tagged five green turtles (*Chelonia mydas*) at a nesting site in the central-southern Red Sea and tracked them for 72–243 days. During the inter-nesting period, the turtles showed high site-fidelity, with a maximum home range of 161 km^2^. After the nesting season, the turtles migrated up to 1100 km to five distinct foraging locations in three countries (Saudi Arabia, Sudan, and Eritrea). Movements within foraging habitats were more wide-ranging compared to inter-nesting movements, with home ranges varying between 1.19 and 931 km^2^. The tracking data revealed that the creation of a relatively small marine reserve could protect the critical inter-nesting habitat in the Farasan Banks. The results also highlight the need for multinational collaboration to protect migratory corridors and foraging sites of this endangered species.

## Introduction

Understanding the movement ecology of wildlife is crucial for implementing effective conservation strategies^1^. This is especially true of highly migratory species with complex, ontogenetic patterns of habitat-use, like sea turtles. After hatching, green sea turtles (*Chelonia mydas*) spend their early life stages feeding on plankton in oceanic waters^2^. Older juveniles generally migrate toward coastal waters for foraging^3^. Once mature, sea turtles migrate back to their natal beaches to reproduce, and females nest near this location^4^. An individual female may lay between 2 and 8 clutches per nesting season, and between nesting seasons females will spend 2–4 years foraging^5^. Between nesting attempts within a season, females rest in nearshore waters (known as their inter-nesting habitat)^6^. After the breeding season, turtles will migrate back to their foraging ground^7^, sometimes covering distances of more than a thousand kilometers^8^. Adult sea turtles thus have three major categories of movements for wildlife managers to consider in conservation planning: inter-nesting movements during a nesting season, migration between nesting and foraging areas, and movements within foraging areas.

Five of the world’s seven species of sea turtles have been found in the Red Sea, but only hawksbill (*Eretmochelys imbricata*) and green turtles are known to nest and forage along the coast^9,10^. Turtle movement patterns are relatively understudied in this region, and all published reports on green turtles have focused on nesting females in the north of the basin^6,11–13^, where most of the major green turtle rookeries are aggregated^10^, though turtles are likely nesting in low-density on most of the suitable sandy islands^14^.

Post-nesting migrations of green turtles have been identified for female turtles tagged in the northern Red Sea, including Ras Baridi, Saudi Arabia^13^, and Zabargad Island, Egypt^11,12^. From these telemetry studies, all of the turtles remained within the basin and did not migrate into the nearby Mediterranean or broader Indian Ocean^12,13^. For example, of the 16 turtles tagged at Ras Baridi, 10 stayed in Saudi Arabian waters whereas two migrated to their foraging habitat in Eritrea, one migrated to Sudan, and three migrated to Egypt^13^. Of the four turtles tagged in Egypt, one stayed in Egypt, one migrated to Eritrea, and the remaining turtle’s tag stopped transmitting in the central Red Sea. Interestingly, green turtles tagged in Oman and the United Arab Emirates have been shown to migrate to the southern Red Sea^15,16^, so there is some evidence of connectivity between these regions. However, there has not been any published tracking of the green turtles that nest in the southern Red Sea, so it remains unclear whether that connectivity is bidirectional. In addition, inter-nesting movements of Red Sea green turtles have only been studied in the northern region^6^, and foraging home ranges within the basin have not been investigated at all.

These knowledge gaps create an urgent conservation challenge for green turtles in the face of rapid regional development. Anthropogenic pressures on turtles may soon increase due to planned coastal projects in the Saudi Arabian Red Sea^17^. In particular, several large developments (known locally as “giga-projects”) are currently being constructed, including NEOM, which is proposed to span 460 km of the northern Red Sea coastline, and The Red Sea Project (TRSP), which plans to develop more than 20 islands between the cities of Umluj and Al Wajh^17^. Understanding the migration patterns of sea turtles in this region is important because their routes might have increased risks of anthropogenic threats, including boat traffic, pollution, ghost nets, etc.^18^. Modification or degradation of habitats used by turtles during inter-nesting or foraging periods could disrupt the life cycle of these animals. The aim of this study was to evaluate the inter-nesting and foraging habitat use and the post-nesting migration patterns of green sea turtles in the central-southern Red Sea. This information is important for the conservation planning of sea turtles in the region.

## Materials and methods

**Figure 1:**
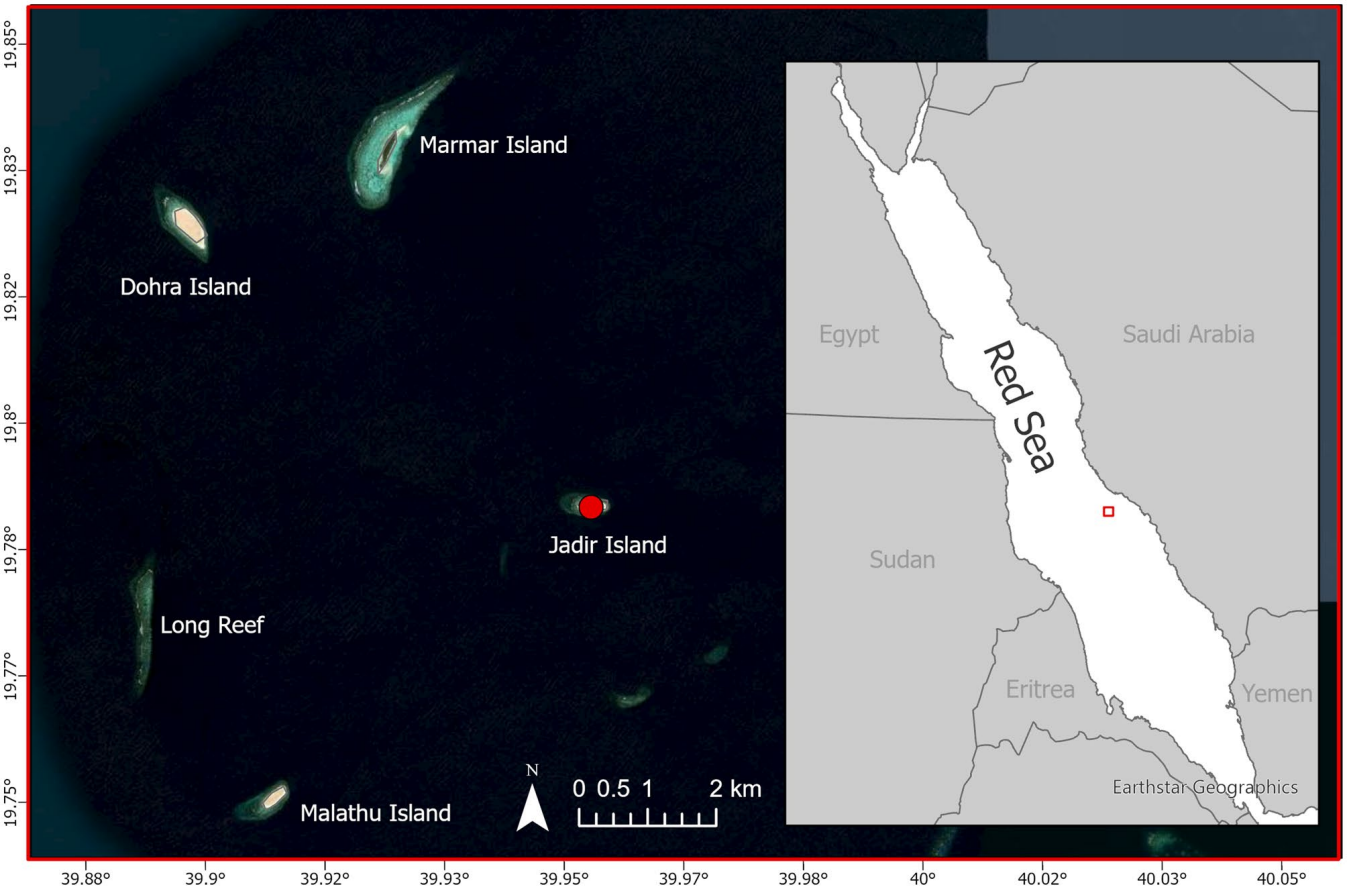
The tagging location of adult nesting female green turtles (*Chelonia mydas*) on Jadir Island, located in the Farasan Banks, 50 km from Al Lith in the Saudi Arabian Red Sea. This small sandy island is within 10 km of Malathu, Dohra, and Marmar Islands, which all show evidence of green and hawksbill (*Eretmochelys imbricata*) nesting. (Satellite image sources: ESRI and Earthstar Geographics on ArcGIS Pro v 3.1 https://pro.arcgis.com/).

Two successful tagging trips were conducted in 2021 on Jadir Island (19.7883°, 39.9546°), 50 km off the coast of Al Lith in the Saudi Arabian Red Sea (Fig. 1). One nesting green turtle was tagged on March 29th, and four were tagged on October 20th, 2021. Jadir Island is approximately 700 m long and 300 m wide, located within 7 km of three other uninhabited islands (Marmar, Dohra, and Malathu). These islands, part of the Farasan Banks complex, are nesting sites for both hawksbill and green turtles^19^. On each survey day, Jadir Island was visited at night to search for nesting female green turtles as they emerged from the sea to lay their eggs. Once the eggs were laid, or if the turtle had a failed nesting attempt and was headed towards the sea, wooden boxes were placed around the turtles to restrain them during the attachment of the satellite transmitter. All tagged turtles were healthy with no major injuries or scars that would impact their ability to successfully nest. The minimum curved carapace length was measured on the midline of the carapace using a flexible measuring tape^20^. Algae and epibionts were removed from the first two ventral scutes of the carapace. Next, sandpaper was used to roughen the area, followed by a final cleaning with acetone. Satellite transmitters (SPLASH10, Wildlife Computers, Washington, USA) were attached to the second vertebral scute of the carapace using the tag attachment kit provided by Wildlife Computers. Finally, after the epoxy had dried, both the satellite tag and the epoxy were coated in marine anti-fouling paint (Hempels, Lundtofte, Denmark). Each turtle was also equipped with two individually-numbered titanium flipper tags (Stockbrands, Perth, Australia), one on the trailing edge of each front flipper. Each turtle was released 2–3 h after initial capture. This work was completed with ethics approval from the KAUST IACUC committee under protocol 19IACUC07. All methods were performed in accordance with the relevent guidelines at the time of the study (e.g., ARRIVE 2.0^21^).

Fastloc GPS-derived locations were manually filtered to remove locations on land and islands. GPS-derived positions with residual error values greater than 35 were also removed^22–24^. The Douglas filter was then applied to the data in Movebank^25^. The hybrid filter was selected, which combines the “maximum redundant distance” filter and “distance angle rate” filter to remove unrealistic locations resulting in swimming speeds > 5 km/h or turning angles > 12.5°^23,24,26^. The home range and core use area of each turtle’s inter-nesting and foraging habitat were calculated separately with the kernel density estimation in R Studio version 2021.09.0 + 351^27^ using the AdehabitatHR package^28,29^ [following^30^]. The calculated *href* was used to generate 95% and 50% utilization distributions (UD) for each turtle using the bivariate normal mode. Turtle 137099 migrated to its foraging ground directly after it was tagged, so it was not included in the analysis of inter-nesting habitat use. The 95% and 50% UDs for inter-nesting and foraging habitats were mapped on ArcGIS Pro v. 2.6.0.

### Ethical approval

Research was conducted under King Abdullah University of Science and Technology (KAUST) Institutional Animal Care and Use Committee (IACUC) approval #19IACUC07.

## Results

**Figure 2:**
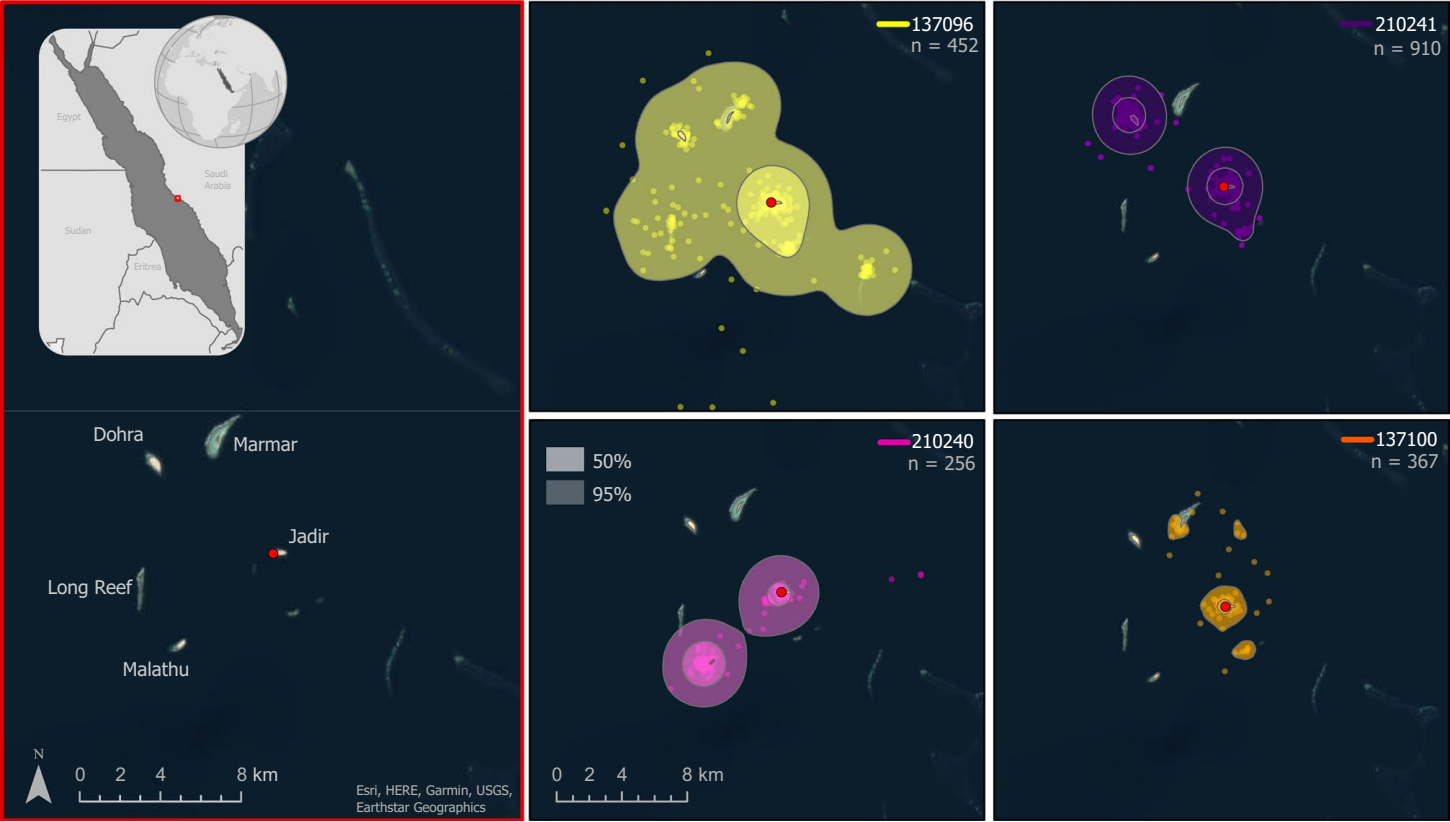
Inter-nesting home range (95% utilization distribution) and core use area (50% utilization distribution) of green turtles (*Chelonia mydas*) tagged while nesting on Jadir Island in the central-southern Red Sea. Each turtle’s Platform Transmitter Terminal (PTT) is indicated in the upper right of the panels, along with the number of transmissions included in the analysis of inter-nesting habitat use. The red dot indicates the location of Jadir, where the tagging took place, and the map on the left shows the names of nearby reefs and islands. The scale is the same on the four panels on the right. (Satellite image sources: ESRI, Earthstar Geographics, and HERE on ArcGIS Pro v 3.1 https://pro.arcgis.com/).

Five female green turtles were satellite tagged while nesting on Jadir Island and tracked for 72–243 days. The tags recorded an average of 959 Fastloc locations (range: 469–1786) (Table 1) and monitored the turtles’ movements during three distinct phases of behavior: inter-nesting, migration, and foraging. Home ranges and core use areas during the inter-nesting phase were small (8.61–160.89 km^2^ and 0.57–18.45 km^2^ respectively) and largely confined to the waters around Jadir and the nearby islands of the Farasan Banks (Fig. 2, Table 2). The tags also recorded five additional nesting attempts by three of the tracked turtles. While most of these attempts occurred on Jadir, two haul-out events were recorded on other nearby islands (Malathu and Dohra), suggesting that the entire archipelago may act as a single rookery. Unfortunately, we were not able to assess nesting success^31^ due to the limited number of haul-out events recorded by tags in our study.

**Table 1:** Collection information of tagged green turtles (*Chelonia mydas*) including individual Argos Platform Transmitter Terminal identifier (PTT), dates the turtle spent in inter-nesting habitat, dates the turtle migrated, the dates the turtle spent in the foraging habitat until the last transmission, number of GPS-derived Fastloc locations, and the location of the foraging ground.

PTT	Dates in inter-nesting habitat	Dates migrating	Dates in foraging habitat	# Fastloc locations	Foraging location
137099	March 29, 2021	March 29–April 8, 2021	April 8–June 09, 2021	477	Dahlak Islands, Eritrea
137096	Oct 20–Dec 10, 2021	Dec 10–Dec 31, 2021	Dec 31, 2021–March 30, 2022	542	Umm Sahar Island, Saudi Arabia
137100	Oct 20–Dec 27, 2021	Dec 27, 2021–Jan 12, 2022	Jan 12–March 8, 2022	469	Ras Baridi, Saudi Arabia
210240	Oct 20–Nov 13, 2021	Nov 13–Dec 21, 2021	Dec 21, 2021–June 20, 2022	1,523	Tiran Island, Saudi Arabia
210241	Oct 20–Dec 30, 2021	Dec 17–Jan 9, 2022	Jan 9–April 22, 2022	1,786	Suakin, Sudan

**Table 2:** Home range (95% utilization distribution) and core use areas (50% utilization distribution) of green turtles (*Chelonia mydas*) tagged on Jadir Island in the Saudi Arabian Red Sea.

PTT	Activity	95% UD (km^2^)	50% UD (km^2^)
210241	Inter-nesting	35.86	7.03
137100	Inter-nesting	8.61	0.57
210240	Inter-nesting	40.79	7.54
137096	Inter-nesting	160.89	18.45
210241	Foraging	5.3	0.72
137100	Foraging	1.19	0.21
137096	Foraging	15.10	1.29
210240	Foraging	5.78	0.79
137099	Foraging	931.01	154.20

Each turtle’s Platform Transmitter Terminal (PTT) is indicated as well as a description of the habitat (inter-nesting or foraging).

**Figure 3:**
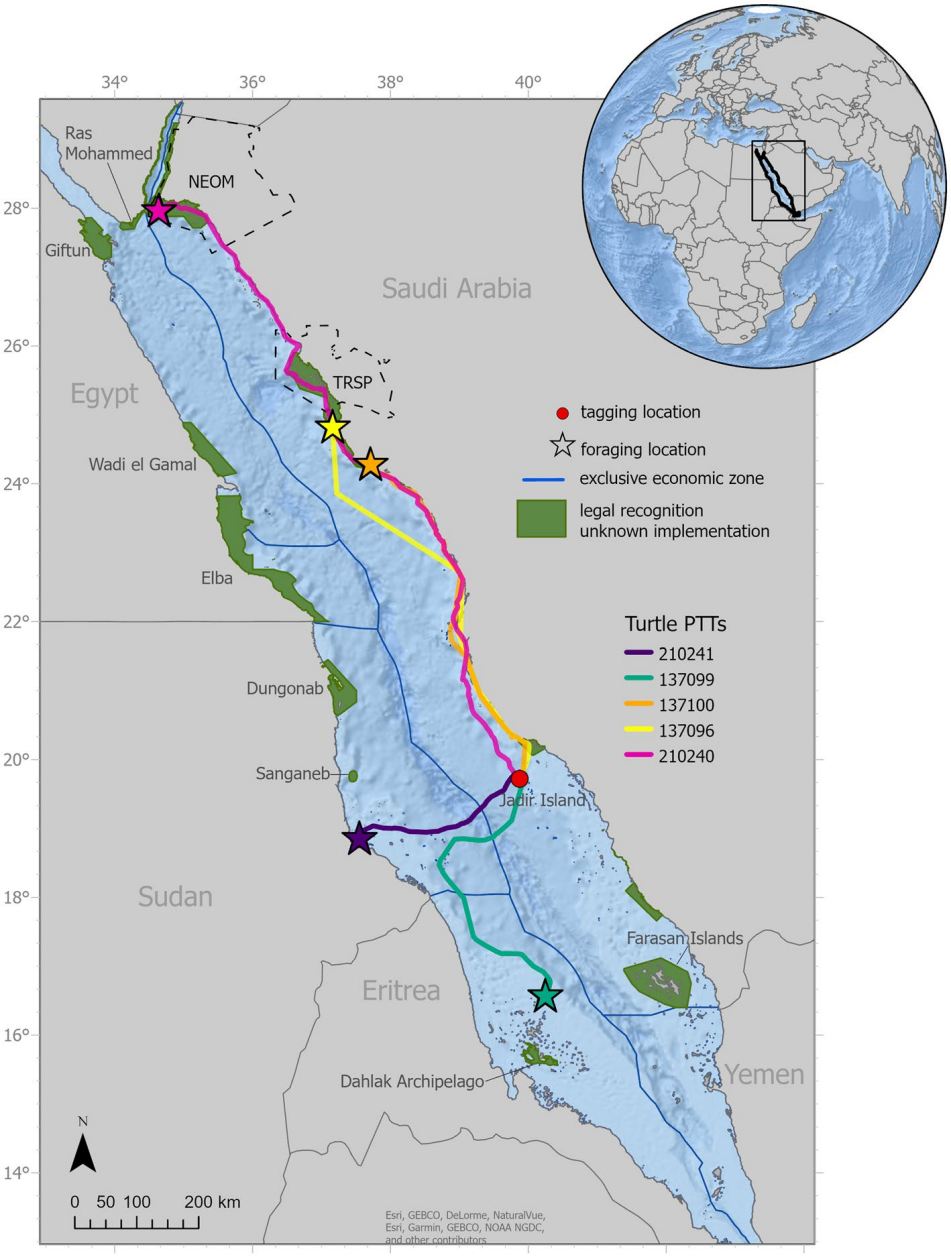
Post-nesting migration patterns of green turtles (*Chelonia mydas*) tagged on Jadir Island (red circle) in the Farasan Banks, 50 km off the coast of Al Lith, Saudi Arabia. Each turtle’s foraging ground is marked by a colored star based on its Platform Transmitter Terminal (PTT). The Red Sea marine protected areas (MPAs) are shaded in green. (Basemap sources: ESRI, JEBCO, Delorme, NaturalVue Garmin, and NOAA ArcGIS Pro v 3.1 https://pro.arcgis.com/).

Each turtle was tracked during their long-distance migrations from the inter-nesting habitat to their respective foraging areas, averaging 628 km (range: 300–1138 km) straight-line distances (Fig. 3). Three of these turtles remained in Saudi Arabian waters for the entirety of the track, each moving north along the same coastal migratory corridor before settling in their respective foraging grounds off Ras Baridi (560 km from Jadir), Umm Sahar Island (660 km), and Tiran Island (1138 km). The two remaining turtles moved into international waters toward foraging grounds in Eritrea and Sudan (Fig. 3). The first of these turtles was tagged on March 29th, 2021, and migrated immediately after tagging, first moving directly west toward Sudan before turning south and settling in Eritrea’s Dahlak archipelago (466 km from Jadir). The second was the only tracked turtle to make multiple stops during a migration. It initially migrated 300 km from Jadir to its foraging ground near Suakin, Sudan. It stayed in Suakin for six days and then migrated back to Jadir where it stayed for a week before returning to Sudan.

**Figure 4:**
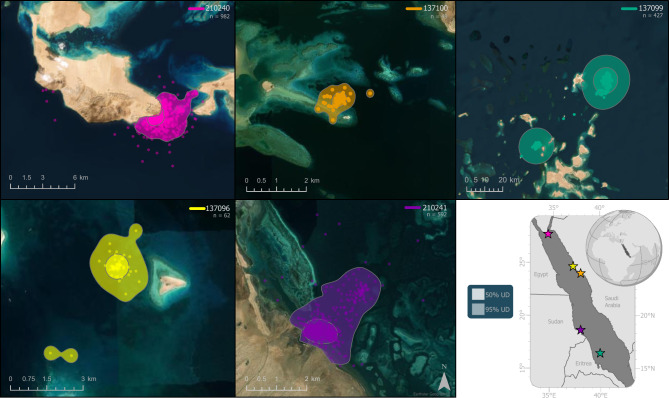
Foraging home range (95% utilization distribution) and core use area (50% utilization distribution) of green turtles (*Chelonia mydas*) tagged while nesting on Jadir Island in the central-southern Red Sea. Each turtle’s Platform Transmitter Terminal (PTT) is indicated in the upper right of the panels, along with the number of transmissions included in the analysis of foraging habitat use. Note varying scale bars on each panel. (Satellite image sources: ESRI and Earthstar Geographics on ArcGIS Pro v 3.1 https://pro.arcgis.com/).

All five turtles migrated to distinct foraging habitats, but the number of transmissions received from these areas varied widely (range: 49–982). The smallest foraging home range (95% UD: 1.19 km^2^) and core use area (50% UD: 0.21 km^2^) also corresponded to the turtle with the fewest transmissions (Fig. 4). Most of the turtles exhibited small foraging areas (95% UD: 5.30–15.10 km^2^, 50% UD: 0.72–1.29 km^2^) (Fig. 4, Table 2), with the exception of turtle 137099, which used a much larger area (95% UD: 931.01 km^2^, 50% UD: 154.20 km^2^). The observed differences in space use did not correlate to either transmission count or to proximity between sites. Rather than being an artifact of limited data, the observed differences in foraging behavior likely correspond to individual variations of the tracked turtles or to differences among their respective grazing habitats.

## Discussion

We tagged five green turtles at one central-southern Red Sea nesting site and they subsequently migrated to five disparate locations spanning nearly the full length of the Red Sea. These results provide new insight into the spatial connectivity between this understudied rookery and other regions of the basin. The tracking data revealed three major categories of movements that can be used by wildlife managers to improve the protection of sea turtles. In particular, we highlight management suggestions for (1) inter-nesting habitat use, (2) post-nesting migratory corridors, and (3) foraging site habitat use.

The inter-nesting ranges of breeding females were constrained (mean 95% home range: 61.54 km^2^) around the nesting island (Table 2). While this is common for the species globally^6,32,33^, work in other regions of the Red Sea showed smaller inter-nesting home ranges (mean 95% home range: 18 km^2^)^6^. Due to the concentrated activity within the critical nesting habitat within the Farasan Banks (including Jadir, Marmar, Dohra, and Malathu Islands), these sites could be designated as a relatively small marine reserve. Although no specific threats have been quantified at this site, our personal observations suggest that Red Sea turtles are subject to many of the same threats as turtles elsewhere (e.g., plastic pollution^34^, fishing-related threats, occasional poaching). Spatial protections could range from no-take mandates^35^, to enforced go-slow zones to reduce the chances of boat-strikes^36^. Gear restrictions on nets, bottom-trawls, and other entanglement/drowning hazards could also reduce bycatch mortality of breeding turtles^37^. Any planned development or construction on these islands should consider the archipelago’s role as a critical rookery for Endangered green turtles and Critically Endangered hawksbill turtles^19^. Additional nesting surveys are also needed to understand more about the abundance and seasonality of turtles nesting in this area, as there is very limited information on these sites.

Of the five green turtles tagged in this study, three remained in the Saudi Arabian exclusive economic zone (EEZ) and transited to their respective foraging grounds through a coastal migratory-corridor. Comparing the data from this study with published literature, the three turtles that stayed in Saudi Arabian waters used a similar coastal migratory-corridor as several post-nesting turtles that were tagged at Ras Baridi, Saudi Arabia^13^, suggesting wide-use among multiple rookeries of Saudi Arabian’s coastal waters. Coastal migratory routes increase the susceptibility of turtles to be impacted by local fisheries that are using shallow, nearshore waters^38,39^. Shallow coastal small-scale fisheries are common along the Saudi Arabian coast^40^, and a study in the Arabian Gulf estimated that 4726 turtles are bycaught annually by artisanal fisheries^41^. In the Saudi Arabian Red Sea, artisanal fisheries are comprised of primarily handlines and gillnets, while industrial fleets use trawl nets and purse seines^40^. The bycatch rates for both the artisanal and industrial fisheries in the Red Sea are unstudied, meaning the threats they pose to sea turtle populations have not been quantified. Future research should look to address this knowledge gap and inform conservation action if necessary.

The remaining two tagged turtles crossed the Red Sea, one of the busiest shipping lanes in the world^42^. The hazard of industrial ship-strikes to local megafauna has been described for other species^43^ and likely poses a similar threat to migrating sea turtles. In addition to the risks of open-ocean migration, transiting through multiple EEZs exposes these animals to different management and protection regimes. Sea turtles and other migratory species would benefit from large-scale multinational protection^1^, which is currently lacking or nonexistent^40^ in this region. All tagged turtles eventually traveled to foraging grounds within the Red Sea corroborating previous population genetic studies^44^, suggesting isolation of turtles using Red Sea rookeries from others within the wider Indian Ocean. Genetic connectivity among Red Sea rookeries has not been studied. Future work should look to assess the population genetics in the center and south of the basin and compare it to previous work from Ras Baridi. This will help conservation planners define the population structure and Regional Management Units for the Red Sea^45^.

Green turtles predominantly feed on seagrasses^46^, so the foraging grounds identified in this study likely correspond to meadows or other grazing habitats^47^. We identified five distinct foraging locations through satellite tracking, which likely does not describe the entire spatial extent of green turtles in the region^48^. This highlights the need for increased tracking efforts in the future to identify more foraging areas, especially in the central and southern Red Sea. Additionally, the foraging home range size (95%) varied widely among tracked turtles (1.19 – 931 km^2^) and this may reflect differences in food abundance and/or density from site to site. One study from the Red Sea^12^ calculated home ranges (95%) of four foraging green turtles (172, 213, 250, and 1095 km^2^) that were similarly variable, but on average were larger than what we found in the present study. It is possible that turtles that forage in patchier areas may need to travel further in order to meet their energetic needs^49^. Similarly, intraspecific competition may increase the energetic cost of foraging in overcrowded meadows. It is difficult to draw general conclusions, however, due to the small number of home ranges that have been measured among these studies. In-water studies, including on foraging ecology, are lacking in the Northwest Indian Ocean^50^. Consequently, it is unclear whether grazing habitats are being fragmented or degraded due to human activities in this region. Further studies can survey the sites identified here to assess the health of each foraging ground and evaluate if there is a correlation between the abundance of seagrass and the turtle’s home range.

Understanding the movement of turtles is critical for their protection. Telemetry, as demonstrated here, is a crucial tool for understanding the habitat use of marine megafauna. The tracked turtles were found to have high inter-nesting site fidelity around Jadir Island, exhibited both coastal and high seas migrations, and had varied space-use patterns at their respective foraging grounds. As discussed here, all of these described behaviors have implications for both future research and management efforts. More work is clearly needed to conserve these vulnerable but valuable species in the face of global change and local human development. This work highlights the need for national-scale protections within Saudi Arabia’s waters and international cooperation with other Red Sea countries to protect migratory species within this young ocean basin^1^.

## Data Availability

The datasets generated during and/or analyzed during the current study will be available by request. Please contact the corresponding author at lyndsey.tanabe@kaust.edu.sa for information.
